# Executive functions and associated brain volumetry in children with persistent stunting and catch-up growth

**DOI:** 10.1038/s41598-025-98238-y

**Published:** 2025-04-22

**Authors:** Beena Koshy, Vedha Viyas Thilagarajan, Roshan S. Livingstone, Manikandan Srinivasan, Venkata Raghava Mohan, Rachel Beulah, Anitha Jasper, Sushil John, Gagandeep Kang

**Affiliations:** 1 Developmental Paediatrics, Christian Medical College, Vellore, Tamil Nadu 632004 India; 2 Department of Community Health, Christian Medical College, Vellore, Tamil Nadu 632004 India; 3 Department of Radiodiagnosis, Christian Medical College, Vellore, Tamil Nadu 632004 India; 4 Wellcome Trust Research Laboratory, Christian Medical College, Vellore, Tamil Nadu 632004 India; 5 Low Cost Effective Care Unit, Christian Medical College, Vellore, Tamil Nadu 632004 India

**Keywords:** Childhood stunting, Catch-up growth, Executive functions, Brain volumetry, Magnetic resonance imaging, Developmental biology, Neuroscience, Neurology

## Abstract

Early childhood stunting can result in sub-optimal executive functions (EF), affecting academic achievements and economic potential in later life. This study hypothesized that children always stunted (AS) at ages 2, 5 and 9 years had lower EF than those who were never stunted (NS). A birth-cohort in Vellore, India was followed up with periodic anthropometric and development/cognitive measures over 2, 5 and 9 years of age. Based on stunting status at these time points, children were classified as NS, stunted at 2 years and caught up by 5 years (S2N5), stunted at 2 and 5 years but caught up later (S5N9), and AS. At 9th year, children underwent neuroimaging using 3T MRI scanner and EF assessment using FAS phonemic fluency test, colour cancellation test and colour trials tests (CTT). From the original birth-cohort of 251, 205 children were reviewed at 9 years. FAS phonemic fluency test showed NS group had significantly higher test scores compared to AS (11.52 vs. 7.4, *p* = 0.02). In CTT, a significant difference in near misses score was observed between NS and AS groups (0.12 vs. 0.38, *p* = 0.03). Upon evaluating unimodal brain association areas, volumes of right occipital fusiform gyrus (9991 mm^3^ vs. 9313 mm^3^; p = 0.04; η^2^ = 0.11), and left lateral occipital cortex (13458 mm^3^ vs. 12559 mm^3^; p = 0.03; η^2^ = 0.07) were significantly higher among NS compared to AS group. Considering higher order association areas, only left pars triangularis was found to be significantly reduced among AS children compared to NS group (4284 mm^3^ vs. 3291 mm^3^; p = 0.01; η^2^ = 0.07). Similarly, there were also significance visible in the basal ganglia regions and the cerebellum. Current study demonstrated EF dysfunction in verbal fluency and inhibitory control in a dose response fashion in groups AS-to-NS with corresponding EF-related brain volumetric changes, highlighting the need for focused nutritional and nurturing approaches in early childhood for gain in human capital.

## Introduction

Around 150 million children worldwide suffer from childhood stunting^1–3^ which can impair their development, cognition, learning performance and subsequent economic and earning potential^4–6^. This influence is related to the effect of undernutrition on brain maturation and development especially in the first 1000 days of life^7,8^. Literature on the associative and predictive effects of childhood stunting has explored predominantly child development, cognition, and school performance including grade achievements^8,9^.

Early childhood stunting and socio-economic status (SES) can influence executive functioning in children which has been proposed as a mediator in lifetime economic potential and achievements^9,10^. Neuropsychological functions including executive functions emerge in mid-childhood and delineate a non-linear and protracted growth pattern influenced by genetics and cultural impacts^11,12^. Executive functions (EF) are higher order skills that help to plan, organise, initiate, monitor and regulate activities and include but not limited to working memory, cognitive flexibility, inhibitory control and planning abilities^13^.

Malnourished children were concurrently shown to have poorer performance on attention and working memory in a cross-sectional study in India^14^. The Young Lives study highlighted the persistent effect of 5-year stunting on EF measured at 12 years which could be a mediator for childhood learning potential^9^. Similarly, children born with low birth weight was shown to have 0.5 SD lower ability on working memory, cognitive flexibility and inhibitory control measured at 4 years of age or later in a recent meta-analysis^15^. Micronutrient deficiency such as iron deficiency in early childhood also had similar persistent effects at 19 years of age^16^. There are contradictory reports. Early childhood associative evaluation of linear growth and EF had shown poor correlation unlike definitive associations between growth, and language, motor and social development in early childhood in a large African birth-cohort^17^.

Neuroimaging correlate evaluation of EF reveals involvement of cerebral frontal and parietal lobes and cerebellar hemispheres^18^. A deep-dive evaluation of neurological structures in childhood stunting is limited as the low-and-middle-income country (LMIC) community settings where stunting is prevalent lack accessible neuroimaging facilities^8,19^. Direct neurological measurements including functional analyses can help to understand evolution of cognitive brain development and possible early childhood influences. The Barbados Nutrition Study (BNS) cohort showed that individuals with early childhood malnutrition had EF deficits in late 40s and different evoked related potentials in their childhood^20^.

The objective of the present study was to analyse executive functions (EF) in mid-childhood (ages 9–11 years) with associated brain neuroimaging in a LMIC birth cohort follow-up with existing information about early childhood stunting and catch-up growth. We hypothesised that children who are always stunted had lower EF when compared to those never stunted.

## Methodology

The present study represents an extension of the follow-up evaluation of an Indian birth cohort: the “Malnutrition and Enteric Diseases” (MAL-ED) cohort. This study was conducted in Vellore, situated in the state of Tamil Nadu, South India, and involved the longitudinal follow-up of 251 children at the ages of 2, 5 and 9 years of age. Further comprehensive details of the MAL-ED study can be found elsewhere^21–23^. This study incorporated anthropometric measures, and psychological assessments of EF, and neuroimaging analyses from the original investigation. At each level of the follow-up, child was enrolled after written informed consent from parents, with additional child assent at 9 years. Parents had the option to withdraw their children from the study at any time and to decline any tests that they found uncomfortable. In the days leading up to the MRI assessments, families received comprehensive information and preparations with the consent of both the parents and the child. The Institutional Review Board and Ethics Committee of Christian Medical College Vellore, India approved the original birth cohort study and subsequent follow-ups.

### Anthropometric measures

For infants up to 2-years old, lengths were assessed utilizing an infantometer, while height measurements post the 2-year mark were taken using a stadiometer. All measurements were rounded to the nearest centimetre. A more comprehensive exposition on measurement procedures, standardization, and post-assessment protocols can be located in previous publications^5,23^. The Multicentre Growth Reference Study (MGRS) standards were employed for data points corresponding to ages 5 and below, and the WHO AnthroPlus software was utilized for information collected at age 9 to compute z-scores for height-for-age and assess stunting^5^.

### FAS phonemic fluency test

The FAS Phonemic fluency test under the NIMHANS neuropsychological battery allows for quantitative assessment of various EF processes based on the total score, including working memory, inhibition and cognitive flexibility^14,24^ and has demonstrated strong inter-rater reliability and good internal consistency^25^. The test was administered by a single psychologist to children, who were instructed to articulate as many words as possible beginning with a syllable within a minute. The psychologist was blinded to the child’s stunting and catch-up growth status to minimise bias. If children attended English medium school, they were asked to generate words beginning with F, A and S and children attending Tamil medium school or local schools asked to generate words beginning with Ka, Pa, and Ma in line with NIMHANS neuropsychological testing battery^14^. The scores were derived from the number of words articulated.

### Colour cancellation test

The Colour Cancellation Test (CCT) is utilized to assess concentration, selective attention, and reading challenges in children^14^. This evaluation, which is part of the NIMHANS Neuropsychological battery for children, requires the child to identify and mark yellow and red circles from a page filled with various colours^11^. The test quantifies and documents the time taken to complete the task (in seconds), the number of omissions (circles not marked in the specified colours), and the number of commissions (circles marked with a colour different from the specified ones).

### Colour trails test-1 & 2

The Colour Trails Test (CTT) was developed to evaluate efficient visuomotor tracking while minimizing the influence of language^26^. Both CTT-1 and CTT-2 demonstrate satisfactory test–retest reliability for completion time variables and appropriate interference test–retest reliability^27^.

During the administration of CTT-1, the psychologist guides the child through a practice trial in which the numbered circles are to be connected in numerical order. If the child encounters difficulties in performing the practice trial, the psychologist intervenes to support. Subsequently, the primary test is administered, and the number of near-misses, number sequence errors, time taken to complete the test (in seconds), and prompts (frequency of the psychologist’s intervention) are calculated and recorded.

Similar procedures are followed in CTT-2, where the child is required to connect the numbered circles in both numerical order and alternating colour order. In addition to other scores in CCT-1, colour sequence errors based on the child’s markings are also calculated.

### MRI image acquisition and processing

The MRI scans were conducted using a Siemens Skyra 3T MRI scanner. High-resolution 3D volumetric T1w images were acquired through the application of the magnetization-prepared rapid gradient-echo (MP-RAGE) sequence with specific parameters: Repetition time (TR) = 2350 ms, Echo time (TE) = 1.74ms, Inversion time (TI) = 1100ms, non-selective excitation at 7°, Field of view (FOV) = 256 mm, and slice thickness = 1 mm. These parameters adhere to the recommended protocols for use with Freesurfer version 6 (https://www.nmr.mgh.harvard.edu/~andre/FreeSurfer_recommended_morphometry_protoco ls.pdf). Pre-segmentation, the visual inspection of all images for quality, artifacts, and clinical abnormalities was performed using the Freeview tool,. Qoala-T, a supervised learning tool, was also used for assessing the quality control of visual scans^28^, after which the images were motion corrected, normalised, skull stripped and parcellated into 68 regions based on the Desikan Killiany atlas using FreeSurfer version 6 software. For the current analysis, details about unimodal and higher order association brain regions were taken from published literature^29,30^The participants during the MRI acquisition process were guided by the project psychologist. Children underwent MRI brain scans without the use of anaesthesia. In cases where children displayed nervousness during the initial appointment, they were scheduled for a second MRI scan. If children remained anxious during the second appointment, they were administered a single dose of Triclofos syrup, not exceeding 15 ml (1500 mg), at a rate of 0.5 ml (50 mg)/kg. The MRI procedure was then carried out while these children were asleep.

### Statistical analyses

In accordance with preceding research, children were categorized into 4 distinct groups based on their stunting status at ages 2, 5 and 9 years, denoted as follows: (i) Group NS: Children who were never stunted, (ii) Group S2N5: Children stunted at 2 years but displaying catch-up growth by age 5, (iii) Group S5N9: Children stunted at 2 and 5 years but exhibiting catch-up growth by age 9, (iv) Group AS: Children stunted at 2, 5 and 9 years^5^.

Multivariate Analysis of Variance (MANOVA) was utilized to ascertain the presence of statistically significant differences in FAS Phonemic Fluency test, CCT, and CTT-1 and 2 scores between the stunted and healthy groups, with adjustments for age and sex based on pre-identified clinical and demographic measurements. Moreover, a comparison of EF-related brain regions was conducted using a separate MANOVA, with age and sex as covariates. To assure independence of observations, each participant’s data was inspected for repeated measures within the same subject.

The comprehensive results of the analyses incorporated beta coefficients and 95% confidence intervals. Our analysis was facilitated using Stata version 17 software, and adjustments for multiple comparisons were implemented by employing the Benjamin-Hochberg procedure, due to its suitability with multiple correlated tests and controlling for false discoveries^31^. Furthermore, the impact of stunting on various brain regions was implemented utilizing Eta squared (η^2^), a widely adopted standardized estimate of effect size in ANOVA models. The effect size interpretation follows Cohen’s guidelines where η^2^ = 0.01 represents a small effect, η^2^ = 0.06 a moderate effect, and η^2^ = 0.14 a large effect^32^.

## Results

In the study, 251 children were initially enrolled at birth, with 46 participants relocating, resulting in a follow-up of 205 participants until the age of 9 years. There was disruption in the 9-year recruitment due to the Covid-19 pandemic. The study completed all cognitive and EF assessments by April 2021 and neuroimaging by December 2021. The MAL-ED India cohort was population representative, and subsequent follow-ups did not differ from the original birth-cohort characteristics and are already published^5,33^.

Of 205 children available at 9 years, 14 participants declined MRI and 6 did not cooperate, leaving 185 children who underwent successful MRI. 12 children among them received triclofos syrup to aid sedation. Ultimately, data from 178 participants were included, as data from 7 children were excluded due to artifacts (*n* = 1), incomplete data (*n* = 2), and failure to fit into any stunting/catch-up categories (*n* = 4). Additional demographic and enrolment details were previously addressed in other studies elsewhere^5,23,34^. Out of 178 participants included in the current analysis, 22 were never stunted. Among the remaining participants, 96 experienced stunting at 2 years but achieved catch-up growth by 5 years. Additionally, 31 participants were stunted at both 2 and 5 years but had catch-up growth by 9 years. The remaining 29 participants were stunted at all time points^5^. Details of MAL-ED India cohort 9-year follow-up are already published^5,35^.

### Executive function related assessments

The comparison of scores from various EF-related assessments is detailed in Table 1. Regarding the FAS Phonemic Fluency Test, the overall score was significantly lower in the AS group compared to NS group (7.4 vs. 11.52 respectively, *p* = 0.02). A similar difference was not seen in groups with catch-up growth.

**Table 1:** Statistical analysis of executive function tests across stunting groups.

	Group NS	Group S2N5	*P*-Value	Group S5N9	*P*-Value	Group AS	*P*-Value
FAS Phonemic Fluency Test							
Overall Score	11.52 (10.25–12.8)	10.35 (8.19–12.54)	0·45	9.79 (7.6-11.98)	0·18	7.4 (4.69–10.12)	**0·02**
F(Ka)	4·46 (3.9–5.01)	3.76 (2.81–4.7)	0·23	3.62 (2.67-4·56)	0·19	2.72 (1.54–3.89)	**0·02**
A (Pa)	3·5 (3·01–3·99)	3.7 (2·86 − 4·54)	0·59	3.51 (2.67–4.35)	0·86	2.33 (1.29–3.38)	0·07
S (Ma)	3·57 (3.13-4·01)	2.89 (2·14 − 3·64)	0·19	2.66 (1.91–3.41)	**0·04**	2.35 (1.42-3·28)	**0·04**
Colour Cancellation Test							
Time in Seconds	90.89 (85.08–96.71)	86.87 (76.89–96.85)	0.61	94.98 (84.82-105.14)	0.34	95.89 (83.2-108.58)	0.54
Number of Omissions	3.52 (2.57–4.46)	5.04 (3.42–6.66)	0.2	2.66 (1.01–4.31)	0.33	2.34 (0.29–4.41)	0.32
Number of Commissions	1.1 (0-2.2)	0.69 (-1.19-2.58)	0.73	0.14 (-1.78-2.06)	0.6	0.17 (-2.22-2.57)	0.65
Colour Trails Test − 1							
Time in Seconds	54.66 (49.32–59.99)	51.03 (41.89–60.17)	0.59	61.38 (51.72–71.03)	0.25	50.57 (39.21–61.93)	0.72
Number Sequence Error	0.18 (0.08–0.28)	0.19 (0.03–0.36)	0.9	0.11 (-0.06-0.29)	0.67	0.13 (-0.08-0.33)	0.65
Near Misses	0.12 (-0.02-0.26)	0.31(0.07–0.55)	**0.05**	0.36 (0.1–0.61)	**0.02**	0.38 (0.08–0.68)	**0.03**
Prompt	1.39 (0.97–1.81)	1.8 (1.08–2.52)	0.32	1.84 (1.08–2.6)	0.26	1.28 (0.39–2.17)	0.9
Colour Trails Test − 2							
Time in Seconds	95.42 (87.68-103.16)	92.23 (78.98–105.5)	0.98	96.69 (82.68–110.7)	0.69	99.97 (83.49-116.45)	0.43
Colour Sequence Error	0.51 (0.31–0.7)	0.81 (0.47–1.14)	0.08	0.48 (0.13–0.83)	0.94	0.75 (0.34–1.16)	0.24
Number Sequence Error	0.1 (0.04–0.17)	0.1 (-0.02-0.2)	0.99	0.02 (-0.09-0.14)	0.4	0.07 (-0.07-0.21)	0.94
Near Misses	0.1 (0.02–0.19)	0.01 (-0.13-0.16)	0.23	0.12 (-0.03-0.27)	0.9	0.03 (-0.15-0.2!)	0.38
Prompt	1.77 (1.36–2.18)	1.54 (0.8402.25)	0.68	1.98 (1.24–2.72)	0.29	1.71 (0.83–2.58)	0.95

Comparison of mean FAS Phonemic fluency test, Colour Cancellation test, Colour Trails Test 1 and 2 scores between the groups was carried out using ANOVA by keeping age and sex as co-variates, with 95% confidence interval. Abbreviations: NS – Never stunted, S2N5 – Stunted at 2 years and caught up by 5 years, S5N9 – Stunted at 2 and 5 years and caught up by 9 years, AS – Always stunted, L= Left, R= Right. Bolded values have significant p-values.

Evaluating sub-domains of FAS-phonemic Fluency Test, individual score related to F(Ka) exhibited significant decline in the AS group in comparison to the NS (2.72 vs. 4.46 respectively, *p* = 0.02). Similar significant distinction was seen with S(Ma) fluency for AS and NS groups(2.35 vs. 3.57 respectively, *p* = 0.04) and S5N9 and NS groups (2.66 vs. 3.57 respectively, *p* = 0.04) All groups performed comparably in the A (Pa) fluency test.

Conversely, the results of the CCT did not yield any significant differences between the groups. However, in the CTT 1 the Near-Misses score was significantly higher in the AS group compared to the NS one (0.38 vs. 0.12 respectively, *p* = 0.03) with a dose response in S2N5 (0.31) and S5N (0.36) groups. None of the other tests including colour sequence error, number sequence error, near misses and prompts in CTT-2 showed any significant distinction.

### Brain volumes

Unimodal Association Areas: Table 2 provides information about unimodal association areas that showed differences in the analyses. Upon evaluating the unimodal association areas, that are responsible for processing information from only one sensory modality, significant group differences were observed in the volumes of right Occipital Fusiform Gyrus (9991 mm^3^ vs. 9313 mm^3^; *p* = 0.04), and left Lateral Occipital Cortex (13458 mm^3^ vs. 12559 mm^3^; *p* = 0.03) between Group NS and Group AS respectively. A dose-response effect due to stunting was visible in the bilateral Lateral Occipital Cortex areas (η^2^, Left = 0.07; Right = 0.11). Other areas did not show any significant differences.

Higher Order Association Areas: Only the left Pars Triangularis showed a significant volume gain in the NS group compared to the AS group (4284 mm^3^ vs. 3291 mm^3^ respectively; *p* = 0.01), with a dose-response effect (η^2^ = 0.07) (Table 2). No other higher order association areas involved in EF showed statistical significance between Groups AS and NS.

Basal Ganglia Regions: Significant volume gains were noticed in areas of Right Pallidum (1705 mm^3^ vs. 1581 mm3; *p* = 0.01), bilateral Caudate (Left: 3488 mm^3^ vs. 3267 mm^3^, *p* = 0.02; Right: 3573 mm^3^ vs. 3383 mm^3^; *p* = 0.03) and Right Thalamus (6559 mm^3^ vs. 6157 mm^3^; *p* = 0.01) in the NS group when compared to the AS one.

Cerebellum: Bilateral Cerebellum Cortex volumes (Left: 53709 mm^3^ vs. 50702 mm^3^, *p* = 0.001; Right: 54072 mm^3^ vs. 50463 mm^3^; *p* = 0.0003) and bilateral Cerebellum White Matter volumes (Left: 12093 mm^3^ vs. 11321 mm^3^, *p* = 0.03; Right: 11749 mm^3^ vs. 11073 mm3; *p* = 0.04) were better in the Group NS in comparison with Group AS. Stunting evaluation in the bilateral Cerebellum Cortex volumes also showed a dosage response (η^2^, Left = 0.07; Right = 0.08).

**Fig. 1:**
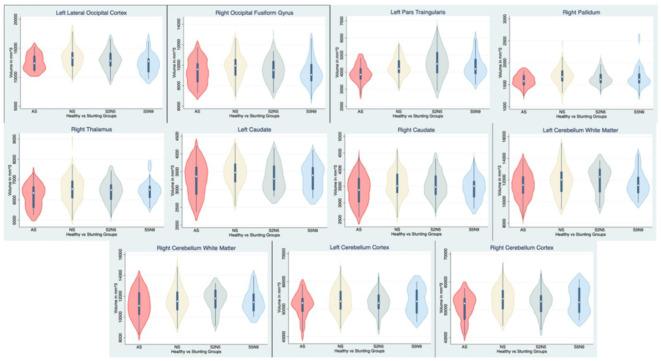
Violin plots of executive function-related brain area volumes exhibiting significance due to stunting and catch-up growth.

The volumes of each region within the EF-related areas that showed significance were compared across the groups through violin plots of all the participants in Fig. 1. This comparison aids in explaining the variation in corresponding brain volumes due to stunting.

**Table 2:** Morphometric analysis of verbal executive function related brain areas across stunting groups.

		Group NS	Group S2N5	*P*-Value	Group S5N9	*P*-Value	Group AS	*P*-Value	Effect-Size
Unimodal Association Areas									
Occipital Fusiform Gyrus	L	9879.44 (9646.22-10112.66)	9319.05 (8919.02-9719.08)	0.02	9427.77 (9001.8-9853.74)	0.14	9557.91 (9083.9-10031.92)	0.3	0.04
	R	9991.07 (9736.79-10245.36)	9544.18 (9108.02-9980.34)	0.1	9226.35 (8761.91-9690.8)	**0.01**	9312.68 (8795.86-9829.51)	**0.04**	0.06
Lateral Occipital Cortex	L	13457.53 (13114.8-13800.25)	12915.07 (12327.23-13502.92)	0.13	12267.33 (11641.37-12893.3)	0.01*	12558.66 (11862.09-13255.22)	0.03*	0.07
	R	13860.09 (13499.08-14221.09)	13308.09 (12688.89-13927.29)	0.17	12129.89 (11470.54-12789.25)	0*	13074.73 (12341.01-13808.45)	0.05	0.11
Higher-order association areas									
Pars Triangularis	L	4283.94 (4147.57-4420.3)	4559.88 (4325.98-4793.78)	0.05	4388.9 (4139.84-4637.97)	0.72	3921.03 (3643.87-4198.18)	0.01*	0.07
	R	4876.37 (4710.12-5042.63)	4876.47 (4591.31-5161.63)	0.92	4916.35 (4612.7–5220)	0.73	4711.75 (4373.85-5049.64)	0.35	0.01
Basal Ganglia									
Pallidum	L	1790·43 (1744·7-1836·17)	1783·74 (1705·29-1862·19)	0·97	1780·76 (1697·22-1864·29)	0·89	1722·22 (1629·26-1815·17)	0·2	0·01
	R	1705·48 (1664·24-1746·72)	1656·38 (1585·65-1727·12)	0·25	1655·94 (1580·62-1731·26)	0·37	1580·9 (1497·09-1664·72)	**0·01**	0·04
Caudate	L	3488·37 (3403·64-3573·1)	3367·54 (3222·21-3512·87)	0·09	3319·84 (3165·09-3474·6)	0·07	3267·3 (3095·09-3439·51)	**0·02**	0·04
	R	3572·96 (3491·34-3654·58)	3535·31 (3395·31-3675·31)	0·45	3422·86 (3273·78-3571·94)	0·1	3383·25 (3217·26-3549·14)	**0·03**	0·03
Thalamus	L	6594·33 (6468·93-6719·74)	6515·21 (6300·11-6730·31)	0·6	6554·19 (6325·14-6783·24)	0·89	6296·6 (6041·72-6551·48)	0·06	0·02
	R	6559 (6441.78-6676.22)	6421·18 (6220·13-6622·23)	0·28	6435·24 (6221·15-6649·33)	0·57	6156.89 (5918·66-6395·13)	**0·01**	0·05
Cerebellum									
Cerebellum White Matter	L	12,092·6 (11816·95-12368·24)	11,861·61 (11388·82-12334·4)	0·46	11,543·64 (11040·19-12047·09)	0·08	11,321·01 (10760·77-11881·24)	**0·03**	0·04
	R	11,748·84 (11499·79-11997·89)	11,455·43 (11028·26-11882·61)	0·25	11,373·53 (10918·65-11828·41)	0·1	11,072·71 (10566·53-11578·89)	**0·04**	0·04
Cerebellum Cortex	L	53,709·02 (52873·5-54544·53)	51,889·19 (50456·1-53322·28)	0·02*	52,194·36 (50668.34-53720.38)	0·13	50,702·14 (49004·01-52400·27)	0·001*	0·07
	R	54,071·97 (53212·6-54931·34)	52,385·93 (50911·92-53859·94)	0·04*	52,791·24 (51221·64-54360·83)	0·2	50,462·74 (48716·12-52209·36)	0·0003*	0·08

ANOVA was done to compare the mean volume values of different brain areas associated with verbal executive functions between the groups by keeping age and sex as co-variates, with 95% confidence interval. The effect size of stunting on the association areas was determined using Cohen’s d. Abbreviations: NS – Never stunted, S2N5 – Stunted at 2 years and caught up by 5 years, S5N9 – Stunted at 2 and 5 years and caught up by 9 years, AS – Always stunted, L = Left, R = Right. Bolded values have significant p-values. * represents a medium effect of stunting on the particular region while ** represents a high effect.

## Discussion

The current analysis evaluated executive functions and associated brain volumes in children persistently stunted in early- and mid-childhood when compared to those never stunted and those who caught up in stature. Groups NS-to-AS showed corresponding dose response in the words produced, which was significant between groups NS and AS in FAS phonemic fluency test, a measure of semantic and phonemic verbal fluency. Time duration taken for CCT, CTT-1 and CTT-2 did not show any significant difference between groups. Near-misses in CTT-1, a marker of inhibitory control was significantly different in S2N5, S5N9 and AS, when compared to NS. Evaluating corresponding brain structures, right Occipital Fusiform Gyrus and left Lateral Occipital Cortex in the unimodal association areas were smaller in the AS group in comparison with the NS group. Among the higher order association cortical areas, left Pars Triangularis was smaller in the AS group. Cerebellar grey and white matters showed smaller volumes along with smaller bilateral Caudate, right Pallidum and right Thalamus volumes in the AS group.

Executive functions considered higher order cognitive processing include but are not limited to goal setting, planning, organising, initiating, managing, regulating, and monitoring activities^13^. Like overall cognitive ability, EF abilities also follow a non-linear, dynamic and inter-dependent evolution in childhood, adolescence and young adulthood, often influenced by environmental factors including cultural experiences, SES factors and learning opportunities^11–13^. Already published literature from this birth cohort showed that groups AS-to-NS showed a significant dose response in verbal, and total cognition scores at 9 years of age with NS children better by 4·6 points (0.3SD) in comparison to AS children indicating persistent effects of early childhood stunting and benefits of catch-up growth in childhood^5^.

The present evaluation on EF adds further evidence that early childhood stunting and catch-up growth show dose response effect on verbal fluency (as evidenced on FAS phonemic test) and impulse or inhibitory control (as evidenced by near-misses on CTT-1). In addition, some cortical, sub-cortical and cerebellar structures controlling EF have smaller volumes in AS children in comparison to NS children indicating brain volumetric effects of early childhood stunting.

Studies in Peru and Ethiopia under the Young Lives Study found that 5-year stunting was negatively associated with working memory and inhibitory control at 12 years of age^9^. In the Barbados Nutrition Study (BNS), adults who had protein energy malnutrition (PEM) in early childhood had poorer inhibitory control at ages 45–51 years^20^. This poor inhibitory control is seen in one of the tests in the current analysis as well (CTT-1). Other tests including CCT and CTT-2 did not show similar association. Our analysis at 9 years included anthropometry, cognition, behaviour and resilience assessments along with EF testing. It is possible that children might have been fatigued as we did EF assessments after cognitive testing.

A meta-analysis of EF in children born preterm or with low birth weight showed that these children (aged above 4 years) performed lower than their peers in core EF, which remained stable over childhood and showed no improvement over time despite better neonatal care in recent years^15^. Chronic severe iron deficiency noted in infancy was negatively associated with inhibitory control, planning and memory tasks at 19 years of age in a long-term follow-up study^16^. It is possible that early childhood risks including but not limited to preterm birth, perinatal events, chronic malnutrition and micronutrient deficiency affect brain development and maturation in the first 1000 days of life causing persistent effects in cognitive processing including that of EF. There can be deficits in structural and functional pathways.

An Indian cross-sectional analysis had shown that malnutrition in childhood was negatively associated with EF probably due to the effect of nutritional deficiency on the development and maturation of these higher order skills^14^. Another Indian study cross-sectionally evaluating individuals 6–23 years old showed that different components of EF can show different maturational levels – stabilization was shown in the order of working memory, inhibitory control and cognitive flexibility and was influenced the most by SES^12^. Meta-analysis evaluating EF’s relation with SES has shown a significant small-medium size relationship between SES and executive function^10^.

There are contradictory reports. Linear growth analysis done in different countries in Africa in infancy showed that growth patterns were associated with motor, language and personal social domains of child development, but not with EF^17^. EF abilities are higher order skills expected to evolve during childhood and are difficult to measure and quantify in infancy, probably explaining the lack of association in the above study^13^.

Concurrent neuroimaging analysis in children with stunting along with EF analysis is limited – neuroimaging analysis in malnutrition itself is considered as a research gap^8,19^. A recent meta-analysis has highlighted that the common finding seen in malnourished children is overall volume loss including cerebral atrophy^36^. A detailed volumetric analysis from our MAL-ED cohort has shown overall total brain volumes, subcortical volumes, bilateral cerebellar white matter and posterior corpus callosum showed a declining dose response in groups NS-to-AS indicating sub-optimal neuronal development in undernutrition in early childhood with partial improvements with subsequent catch-up growth^35^. In the BNS study, children who had PEM in the first year of life had shown increased paroxysmal and focal abnormal electroencephalographic changes at 5–11 years of age, with further evaluations showing EF dysfunction in adulthood^20,37^.

Systematic review of neuroimaging associations of EF showed that frontal, parietal and cerebellar lobes were associated with EF scores^18^. Both cerebellar cortex and white matter had smaller volumes in AS children in our analysis also. Cerebellum in addition to its role in motor control has influence in executive, emotional and social functions with varying roles during lifespan – crucial role in infancy and childhood with a more supportive role in adulthood as cortical structures mature^38,39^. It is also a network/integrating/co-ordination centre and integrates with cortical structures, basal ganglia and peripheral systems^39^. Basal ganglia especially putamen and caudate structures augment cognitive-motor interactions through networking connections and neurotransmitters such as dopamine^40^. In addition to networking, basal ganglia helps in complex data selection, relaying relevant information and inhibiting interfering activities by increased integration of internal brain structures and external sensory inputs^41^.

Pars triangularis, the frontal epicentre of semantic and lexical processing is smaller in the AS group in comparison with the NS group in our study^42^. It is possible this underlying structural deficit has caused phonemic fluency impairment as seen in the current analysis. The prefrontal cortex, the neurological anchor of EF, matures in late adolescence extending to the third decade, probably explaining the sparing effect seen in childhood stunting as seen in our study^43^. Unlike the systematic review of neuroimaging and EF association which highlighted the absence of occipital lobe usually in EF functions, our study showed that left lateral occipital cortex and right occipital fusiform gyrus were smaller in AS group when compared with NS^18^. Lateral occipital cortex and occipital fusiform gyrus are involved in visual stimulus processing, visual awareness emergence and audio-visual integration^44^. The larger fusiform gyrus is considered important not just for visuo-auditory integration and higher order visual abilities but also for functional processing especially in perceptual skills^45^. Most areas in the brain involved in EF other than the illustrated ones did not show any significant differences in this cohort highlighting the role of possible compensatory mechanisms.

Early childhood nurturing care and learning opportunities along with optimum nutrition can optimise some of the deficits we have seen in this analysis. The integrated child development services (ICDS) of government of India have foreground the importance of nurturing care framework and early childhood education along with nutritional supplementation in their Anganwadi centres through the flagship program – *“Poshan bhi*,* Padhai bhi”* (learning along with nutrition). Appropriate early childhood support along with school level learning aids can alleviate some of the EF dysfunction in stunting as seen in this study.

Uniqueness of the present analysis include EF evaluation in a birth cohort group of children with varying stages of stunting and catch-up growth with associated neuroimaging assessment. Main strengths include the sample size, long-term follow-up, availability of quality data, EF assessment using culturally appropriate measures and neuroimaging. The Covid-19 pandemic caused disruption to the recruitment in 2020–2021. Anthropometric, cognitive and neuroimaging analysis are adjusted for age to address age disparities. Other limitations include the current analysis of only one LMIC site in urban Vellore, which could affect generalisability to other geographically and culturally diverse settings. It also should be highlighted that EF is an evolving dynamic concept in childhood and even specific testing in mid-childhood need more robust psychometric data^46^. Being a LMIC cohort, other potential confounders could have included genetic, epigenetic and antenatal influences, which are not explored in the current analysis.

## Conclusion

This unique analysis in a birth-cohort follow-up study highlighted EF dysfunction in verbal fluency and inhibitory control in a dose response fashion in children persistently stunted and those caught-up in linear stature in comparison to children never stunted. Brain volumetric analysis indicated impairments of occipital association areas, pars triangularis, subcortical structures of basal ganglia and cerebellum, all of which can be involved in EF at this age. Being a first report, additional studies are required to validate these findings and to explore macronutrient and micronutrient supplementations along with responsive and nurturing caregiving in early childhood as potential interventions to improve EF. Further research also can expand current findings to understand possible causative pathways, compensatory mechanisms, and pathophysiology to modify these possible interventions in early childhood.

## Data Availability

All MAL-ED data till 5 years is available at https://clinepidb.org/ce/app . The anonymized version of the MAL-ED Data including MRI Brain parcellation data is available in the Harvard Dataverse database. Below is the link to our data: https://doi.org/10.7910/DVN/KJGMAY.
